# Mapping the operational landscape of microRNAs in synthetic gene circuits

**DOI:** 10.1038/s41540-017-0043-y

**Published:** 2018-01-11

**Authors:** Tyler Quarton, Kristina Ehrhardt, James Lee, Srijaa Kannan, Yi Li, Lan Ma, Leonidas Bleris

**Affiliations:** 1 Bioengineering Department, University of Texas at Dallas, Richardson, TX USA; 2 Center for Systems Biology, University of Texas at Dallas, Richardson, TX USA; 3 Department of Biological Sciences, University of Texas at Dallas, Richardson, TX USA; 4 School of Behavioral and Brain Sciences, University of Texas at Dallas, Richardson, TX USA

## Abstract

MicroRNAs are a class of short, noncoding RNAs that are ubiquitous modulators of gene expression, with roles in development, homeostasis, and disease. Engineered microRNAs are now frequently used as regulatory modules in synthetic biology. Moreover, synthetic gene circuits equipped with engineered microRNA targets with perfect complementarity to endogenous microRNAs establish an interface with the endogenous milieu at the single-cell level. The function of engineered microRNAs and sensor systems is typically optimized through extensive trial-and-error. Here, using a combination of synthetic biology experimentation in human embryonic kidney cells and quantitative analysis, we investigate the relationship between input genetic template abundance, microRNA concentration, and output under microRNA control. We provide a framework that employs the complete operational landscape of a synthetic gene circuit and enables the stepwise development of mathematical models. We derive a phenomenological model that recapitulates experimentally observed nonlinearities and contains features that provide insight into the microRNA function at various abundances. Our work facilitates the characterization and engineering of multi-component genetic circuits and specifically points to new insights on the operation of microRNAs as mediators of endogenous information and regulators of gene expression in synthetic biology.

## Synthetic biology: microRNA-based regulation

MicroRNAs are increasingly important regulatory modules in genetic circuits. To probe their versatile nature, a systems and synthetic biology research team led by Dr. Leonidas Bleris at the University of Texas at Dallas, combined experiments and mathematical modeling to study the microRNA operational landscape. The team engineered custom genetic circuits that contain microRNA-based regulation and introduced an analytical strategy, that includes clustering and superposition of discrete experiments, to produce a “bird’s-eye view” perspective. The team then developed mathematical models with informative features and predictive value. One feature highlighted in this work correlates the function of a microRNA to its concentration and the genetic circuit abundance. The results of this work may help towards rationally utilizing microRNAs in engineered gene circuits.

## Introduction

MicroRNAs (miRNAs) are endogenously expressed in animals, plants, and viruses and regulate the expression of nearly 30% of all protein-coding genes.^1,2^ The means by which miRNA mediate protein synthesis primarily depends on the degree of complementarity between the seed sequence of the miRNA and its target, located in messenger RNAs (mRNAs).^3,4^ Once processed, a mature miRNA embedded within an RNA-induced silencing complex (RISC) guides the complex to a mRNA and binds to its corresponding target through Watson–Crick base pairing.^5^ In the case of perfect or near perfect complementarity as observed in plants, the endonucleolytic activity of an Argonaute protein residing within the RISC complex initiates resulting in the cleavage of the mRNA transcript through the RNA interference (RNAi) pathway.^6,7^ In animals, the vast majority of miRNA-guided RISC complexes bind to partially complementary mRNA target sequences. This partial complementarity still causes mRNA destruction through a variety of means including the blocking of translation initiation, recruitment of translation blockers, the deadenylation of the 3′ untranslated region, and/or the decapping of the 5′ untranslated region.^8^ Quantitative proteomic profiling in human cells reinforced the view that the majority of all miRNA-based protein regulation in mammals was due to mRNA destabilization^9^ whereas only 11–16% of miRNA-based regulation was shown to be caused by ribosomal translational efficiencies.^10^

Gene networks have evolved to be able to exploit their stochastic environment to their operational advantage^11^ where miRNA are thought to have important role in conferring robustness to endogenous processes.^12^ Mathematical models that probe the properties of miRNA regulation have unveiled a collection of nuanced regulatory effects.^13–15^ Experimentally, gene circuits equipped with endogenous miRNA sensors demonstrate that both perfectly and partially complementary miRNAs reduce noise for low expression genes, but increased noise for high expression genes.^16^ Regulatory miRNA-based feedforward and feedback loops, frequently observed motifs in mammalian cells,^17^ are shown to provide genetic template adaptation,^18^ dosage compensation,^19^ and noise buffering^20,21^ properties. In addition to the more subtle aforementioned network regulatory effects, miRNA that participate in positive feedback loops have been show to directly control the p53-MDM2 core^22^ and state switching between epithelial and mesenchymal states in metastasis.^23^ At an additional layer of complexity, the network of miRNAs and their target mRNAs allows for cross-regulation between different endogenous mRNAs by sequestering the shared miRNAs. These sequestering properties are largely determined by the relative abundance and the binding strength of miRNA and mRNAs.^24^

The adoption of RNAi in synthetic biology led to significant advancements in gene circuit functionality.^25–28^ Specifically, engineered synthetic miRNAs are frequently used as regulators in gene circuits.^29–33^ Additionally, gene circuits equipped with passive miRNA sensing elements establish an interface between a computing system and endogenous miRNAs. The first demonstration of an interface between siRNAs and engineered RNAi targets established a universal logic evaluator capable of computing conjunctive and disjunctive normal form expressions with up to five input variables.^34^ Subsequently, endogenous miRNAs were used as inputs in complex sensor/computation architectures that differentiate between cancer and healthy cells.^35^ A molecular diagnostics circuit was engineered to sense both endogenous miRNA and transcription factor abundances and perform logic operations.^36^ There are a number of recent papers that explore a combination of theoretical and experimental avenues to optimize design parameters for genetic circuits operating in cells.^33,37–40^

As a general observation, the relative abundance of miRNA and their associated target mRNA markedly impacts protein repression and genetic circuit functionality. Here, we shed new light on the relationship between input genetic template abundance, miRNA concentration, and output under miRNA control. We use the complete operational landscape of a synthetic gene circuit and we derive a phenomenological model that provides insight into the miRNA function at various abundances.

## Results

**Fig. 1:**
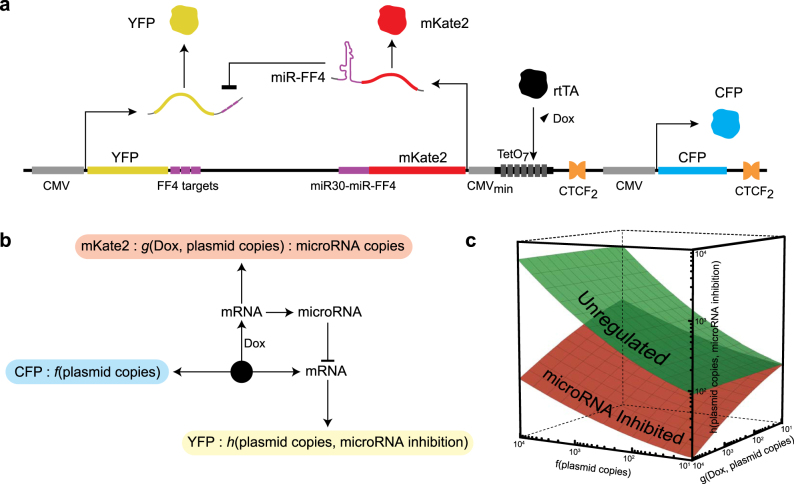
Circuit architecture and function. **a** Biological schematic of the miRNA repression circuit. The CFP fluorescent protein is constitutively produced and is used to quantify the plasmid copy number. The synthetic miRNA-FF4 is produced in response to Dox and is quantified by mKate2 fluorescence. The miRNA-FF4 represses the mRNA of the output fluorescent protein, YFP. **b** Functional graph representation of the circuit. Each observed fluorescent quantity is assumed to be a function of the underlying activity of the circuit elements. **c** Visual representation of function-dependent output space of circuit. The green and red surfaces represent the expected output space of the circuit in the absence and presence of miRNA regulation, respectively

We engineered a custom genetic circuit that consists of the following components (Fig. 1a): (a) an inducible synthetic miRNA co-expressed with an mKate2 fluorescent protein, (b) the constitutively produced fluorescent protein TagYFP (YFP) containing synthetic miRNA targets, and (c) the constitutively produced fluorescent protein TagCFP (CFP). mKate2 and its accompanying synthetic miRNA-FF4^18,41^ are under direct transcriptional control via an inducible TRE promoter. The TRE promoter is activated by the endogenously expressed transcription factor rtTA complexed with a small molecule, doxycycline (Dox). On the same plasmid, we engineered the miRNA-FF4 sensing element by placing three adjacent FF4 targets^34^ in the 3′ untranslated region (UTR) of the fluorescent protein YFP, which is constitutively produced by the cytomegalovirus (CMV) promoter. The plasmid also harbors a constitutively produced fluorescent protein CFP. The CFP levels primarily depend on the plasmid copy number while the mKate2 fluorescence is proportional to miRNA-FF4 levels, as they are produced from the same transcript (Supp. Fig. 1). As a control, we engineered an additional circuit which was an exact replicate of the fully operational circuit in all aspects apart from the exclusion of miRNA-FF4 targets on the 3′ UTR of YFP (Supp. Fig. 2).

The expression of the fluorescent proteins can be viewed as a collection of interdependent functions whose arguments are composed of quantities corresponding to the various abundances of active circuit elements (Fig. 1b). From this perspective, CFP fluorescence is functionally mapped to plasmid copy number, mKate2 fluorescence is a function of both Doxycycline concentration and plasmid copy number, and YFP fluorescence is a function of both miRNA concentration and plasmid copies. From this function-based vantage point, we aim to quantitatively map the complete three-dimensional fluorescent expression space, or operational landscape, of the plasmid’s output with respect to its constituent circuit elements (Fig. 1c) and decouple the respective contributions.

In order to probe the impact of plasmid and miRNA copy number to the expression of the output protein, we partitioned the three-dimensional space generated from flow cytometry measurements of the circuit’s three fluorescent proteins. We quantitatively characterize the impact of plasmid copy number and miRNA concentration on the regulated expression of the output protein by exploring the features resulting from the mapped output surface in this expression space. The approach we employed generated a broadly-bounded expression space with fine-grained resolution which facilitated the development of a descriptive phenomenological model of the circuit output.

We performed a Dox titration (0.001–1 ng/mL) in HEK293 Tet-On (HEK293t) cells which contain a constitutively produced rtTA stably integrated into their genome. Approximately 150 thousand cells were plated per well and were transiently transfected 24 h later. Immediately following transfection, the cells were induced with Dox and were allowed to grow an additional 48 h before obtaining measurements using flow cytometry.

**Fig. 2:**
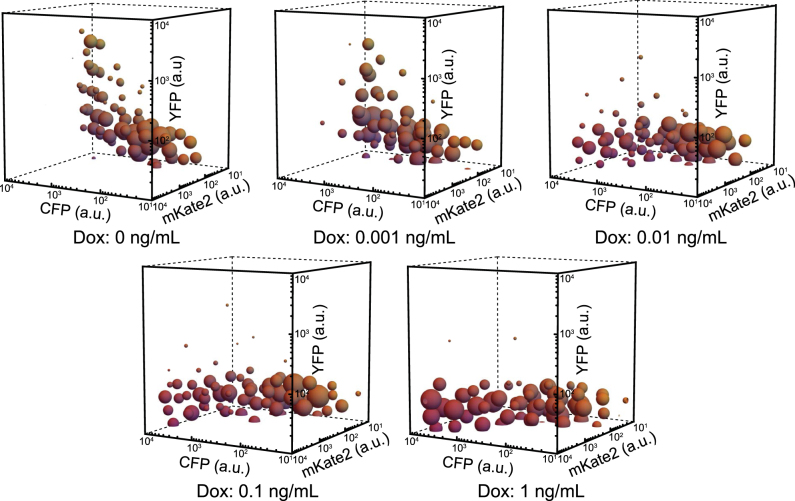
Dox titration cluster plots. Each sphere is a cluster of cells resulting from the *k*-means clustering algorithm. The color of a cluster is a blend of yellow, red, and blue in proportion to the cluster’s mean values of YFP, mKate2, and CFP fluorescent arbitrary units, respectively. The diameter of a cluster is proportional to the number of cells it contains

An initial qualitative assessment of the cytometry data in a three-dimensional fluorescence space for the selected concentrations of Dox generally confirmed our expectations for mKate2 and CFP, where mKate2 was seen to be proportionally expressed with respect to CFP, while also revealing a unique copy number and miRNA concentration dependent YFP (output) expression (Fig. 2) that was not observed in the control (Supp. Fig. 3).

### Pairwise expression relationships

To understand how the combined influences of plasmid copies and miRNA concentration effect the output protein’s expression, we examine the pairwise relationships between each of representative fluorescent signals (Fig. 1c). In order to discretize CFP fluorescence to define the plasmid copies, we partitioned the cytometry data into fixed bins of CFP fluorescence intensity (100 arbitrary units) and statistically averaged the YFP and mKate2 expression values of the cells collected in each bin, respectively. For example, the first bin, whose width corresponds to CFP fluorescent values ranging from 0–100 a.u. collects all the single cell whose observed CFP expression falls within the aforementioned range. These cells, which also express YFP and mKate2, are reduced to a single data point by averaging the observed expressions of YFP and mKate2. This procedure is repeated for each bin of CFP fluorescence with a minimum of 100 cells to ensure reliable statistics. Effectively, this binning process condenses the three-way fluorescence expression of single cell sub-populations into representative data points partitioned with respect to their plasmid copy number.^18^

**Fig. 3:**
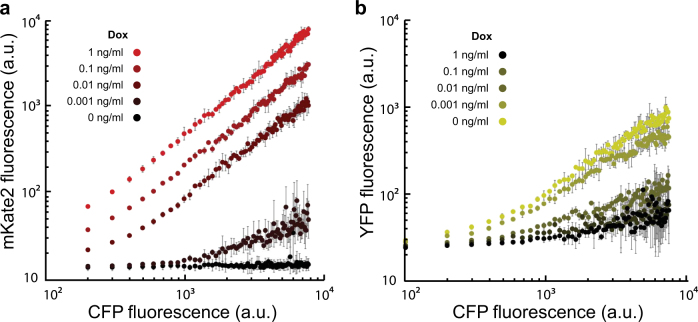
Circuit response. **a** mKate2 fluorescence increases as CFP increases in a Dox dependent manner. **b** YFP has a nonlinear saturation at higher CFP fluorescence where the degree of saturation is Dox dependent. The error bars correspond to the standard deviation of an experimental triplicate

**Fig. 4:**
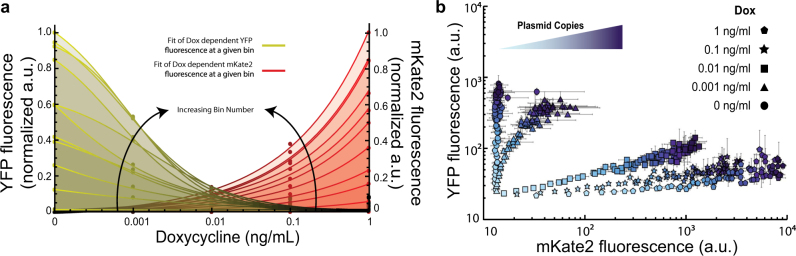
Circuit response. **a** Relative YFP and mKate2 fluorescence in response to Dox. 10, linearly spaced CFP bins ranging from 100 a.u. to 10^4^ a.u. were selected at each Dox concentration for both YFP and mKate2, depicted as dots. The fluorescent values of YFP and mKate2 were then normalized with respect to the highest CFP bin. Bins corresponding to the same CFP fluorescence throughout each Dox concentration were then fit. The relative expression of YFP follows a saturating function where a saturation effect at 0 Dox is observed whereas the relative expression of mKate2 exponentially increases as a function of Doxycycline. **b** YFP is overall suppressed as Dox increases where, at a given copy number, YFP is inversely proportional to mKate2. The error bars correspond to the standard deviation of an experimental triplicate

The binned data confirmed that miRNA-FF4 concentration and plasmid copies share a linear relationship at all Dox concentrations in both the circuit and its control (Fig. 3a, Supp. Fig. 4). We observed the YFP fluorescence trending from an initially uninhibited linear response at 0 ng/mL Dox to a saturating-like response at 1 ng/mL Dox, where at high plasmid copies, the output’s expression becomes increasingly invariant to plasmid copy numbers^18^ (Fig. 3b, Supp. Fig. 5) where a strict linear response was observed in the control (Supp. Fig. 6). The relative expression of mKate2 in each bin grew exponentially as a function of increasing Dox for both the circuit and its control whereas the relative expression of YFP exhibited saturation in the absence of Dox (Fig. 4a, Supp. Fig. 7). Additionally, we confirmed the output decrease in the presence of increasing miRNA concentration, where the output was seen to be inversely proportional to miRNA concentration at a given plasmid copy number (Fig. 4b) as compared to the output’s miRNA independence observed in the control (Supp. Fig. 8).

### miRNA repression efficiency

In order to examine the copy number dependence on miRNA-based output suppression, we compared the relative change of mean regulated output expression of each of the Dox induced cells as compared to the uninduced state at each copy number bin. The above-mentioned binning procedure was applied to both the circuit and its corresponding control.

As an example, the efficiency of miRNA repression at the lowest copy numbers, i.e., the subpopulation of cells which fell within the first bin, was calculated by taking the ratio of the circuit’s YFP value at its first bin to the control’s YFP value at its first bin and subtracting the resulting fraction from 1. In the extreme case, if there was no difference in YFP expression between the circuit and the control, the ratio would equate to 1, which would correspond to an efficiency of 0 and the effects that miRNA had on the circuits output expression at that copy number would be conclusively null. On the other hand, if there was a dramatic difference between the YFP expression of the circuit as compared to the control at that bin, the ratio would be effectively 0 which reflects an efficiency of 1 where the presence of miRNA in the circuits environment had a significant effect on the circuit’s output protein expression.

As such, the efficiency of repression, *ρ*, for each bin was calculated as

$\rho _{b,d} = 1 - \frac{{n^c_{b,d}}}{{n_{b,d}}}\frac{{\mathop {\sum }\nolimits_i^{n_{b,d}} \,i_{YFP}}}{{\mathop {\sum }\nolimits_i^{n^c_{b,d}} \,i_{cYFP}}} - \varepsilon = 1 - \frac{{\mu YFP_{b,d}}}{{\mu cYFP_{b,d}}} - \varepsilon$ where indices *b* and *d* refer to the bin (1, 2, …, 80) and Dox (0, 0.001, 0.01, 0.1, 1 ng/mL) concentrations, respectively, *n* refers to the total number of cells, *i*_*YFP*_ and *i*_*cYFP*_ are the YFP fluorescence values of the circuit and control of cell *i*, respectively, and *ε* is an term which corrects for the absolute difference in expression levels of the circuit and the control, given as the relative difference in expression of the control and circuit of each bin at 0 ng/mL Dox

$$\varepsilon = 1 - \frac{{n^c_{b,0}}}{{n_{b,0}}}\frac{{\mathop {\sum }\nolimits_i^{n_{b,0}} \,i_{YFP}}}{{\mathop {\sum }\nolimits_i^{n^c_{b,0}} \,i_{cYFP}}}.$$

**Fig. 5:**
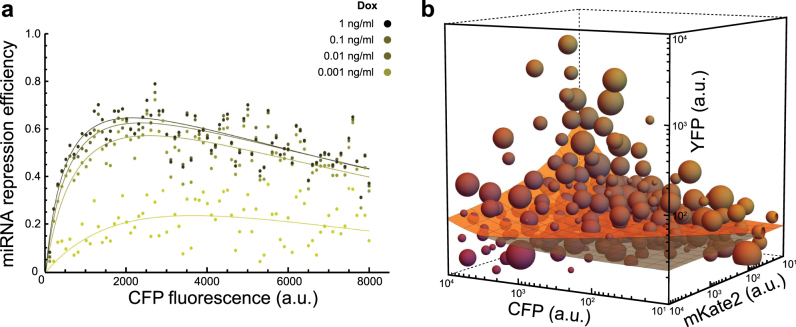
**a** Copy number dependence of miRNA repression efficiency. The percent change of YFP fluorescence was calculated for each bin and non-zero Dox concentrations with respect to the 0 Dox control circuit. The effective repression of YFP follows an exponentially decaying, saturating function where YFP reaches a maximum at plasmid copy numbers corresponding to CFP fluorescent values of ~2000 (a.u.) and exponentially decays as plasmid copy number increases. **b** Operational output space of circuit. Data from all Dox concentrations were superimposed and clustered. The diameter of a cluster is proportional to the number of cells it contains and the color is a proportional blend of yellow, red, and blue with respect to the cluster’s mean YFP, mKate2, and CFP, respectively. The orange lamina represents a proposed relationship of YFP expression as a function of mKate2 and CFP. The gray portion of the lamina along with the bounds of the plot are exclusionary regions where YFP expression was not observed

Initially, miRNA repression efficiency increased as copy numbers increase, reaching a maximum at copy numbers corresponding to ~1000 a.u. for each dox case. As plasmid copy number continues to increase, the repression efficiency gradually decreases (Fig. 5a). The behavior of the repression efficiency, *η*, was best fit with a two parameter exponentially decaying, saturating function, modeled as

1 $$\begin{array}{*{20}{c}} {\eta \left( c \right) = e^{ - \gamma \,c}\frac{c}{{\omega + c}}} \end{array}$$

where *γ* is the decay constant, *ω* controls the rate of saturation with respect to *c*, which represents the plasmid copies. Using Pearson’s correlation, the coefficient of determination (R-Squared) values obtained from the fits for the 1, 0.1, and 0.01 ng/mL Dox concentrations were all above 0.95 where the 0.001 ng/mL Dox induced group had an R-Squared value above 0.8. Interestingly, the decay constant *γ* was found to be the same at all Dox concentrations, thus attributing the differences in behavior of the repression efficiency at different Dox concentrations to variations in the parameter *ω*.

### Complete operational space of circuit

In order to obtain a complete representation of the circuit to capture the collective effects of plasmid copies and miRNA concentration on the output, we superimposed the data collected from all Dox concentrations then condensed the resulting data by employing a k-means clustering^42^ algorithm for both the circuit and its control (Fig. 5b, Supp. Fig. 9). From this perspective, we were able to visualize the circuits operational expression profile at all levels of miRNA concentration and plasmid copies. By coupling the individual pairwise relationship (Fig. 2) along with the uncovered repression efficiency (Eq. 1), we constructed a model whose intention is to represent the complete operational output space of the circuit,*π*, as a function of plasmid copies and miRNA concentration, *μ*, given as $\pi \left( {c,\mu } \right) = \alpha \frac{c}{{\eta \left( c \right)\mu }} + \beta = \alpha \frac{c}{{e^{ - \gamma \,c}\frac{c}{{\omega + c}}\mu }} + \beta$ which simplifies to,

2 $$\begin{array}{*{20}{c}} {\pi \left( {c,\mu } \right) = \alpha \frac{{\left( {c + \omega } \right)}}{{e^{ - \gamma \,c}\mu }} + \beta } \end{array}$$

where *α* is a proportionality constant, *β* is a parameter which represents the basal expression of the output, and *γ*, *c*, and *ω* are the same parameters previously discussed (Eq. 1).

The model is composed of three experimental observations; namely, the output is proportional to plasmid copies (Fig. 3b), inversely proportional to miRNA concentration at a given plasmid copy number (Fig. 4b), and the effect of miRNA concentration is scaled by an exponentially decaying saturating function (Fig. 5a).

The constructed model was then fit to the clustered data in three-dimensional expression space of the superimposed, clustered data with an R-Squared value of ~0.85 (Fig. 5b). Sensitivity analysis of equation (2) revealed that the circuit output is increasingly sensitive to changes in miRNA concentration as plasmid copies increase (Supp. Fig. 10) but increasingly robust to changes in plasmid copies as miRNA concentration increases (Supp. Fig. 11).

## Discussion

Our analytical framework enabled us to uncover a unique, previously unexplored effect which played an important role in shaping the regulated protein’s expression behavior as its genetic template increased that was not obvious using the traditional ODE modeling approach (Supp. Fig. 12). Specifically, the observation that miRNA repression drops in efficiency as plasmid copies increases suggests that the rates of the biological processes which generate the output protein’s mRNA and the miRNA effector do not scale in tandem as functions of plasmid copies. By mapping the complete circuit’s output, we observe that at higher plasmid copies, the output can out produce the miRNA and eventually overcome repression. One possible explanation is that some of the processing elements uniquely responsible for miRNA biogenesis become saturated due to our circuit producing synthetic miRNA in abundances which far exceed physiological concentrations.

This nuanced effect of miRNA repression at higher genetic copy could be present either in naturally occurring copy number variation genomic regions or consequentially through genomic instability. From an engineering perspective, the consequence of our work provides insight into the subtle behavior of plasmid based, transient gene delivered treatments as the genetic load is non-uniformly distributed among the impinged cells. Taking into account the effects we present could also provide a blueprint from which miRNA-based therapeutics would be optimized or utilized the nonlinear repression efficiency for template quantity specific gene suppression.

## Methods and procedures

### Recombinant DNA cloning

The restriction enzymes, polymerase, and T4 ligase enzyme used for cloning and ligation were obtained from NEB. QIAGEN Plasmid isolation, gel extraction and PCR purification kits were used. For transformation, either NEB-5alpha competent *E. coli* (catalog #C2987H) or competent DH5alpha cells (originally obtained from Life Technologies) were prepared using the standard CaCl_2_ method of competent cell preparation. Bacterial culture media and agar (BD Biosciences) were prepared according to manufacturer’s instructions. Primers for the experiments were designed using A Plasmid Editor (Ape–version 1.17) and synthesized from Sigma. The primers received were diluted into stocks of 100 pmol/μL. The plasmid was sequenced by Genewiz.

### Cell culture and transfection

HEK293 TET-On Advanced cells (Clontech, Catalog #630931) were maintained at 37 C, 100% humidity and 5% CO_2_. The cells were grown in Dulbecco’s modified Eagle’s medium (DMEM) (Invitrogen, Catalog #11965–118) supplemented with 10% fetal bovine serum (FBS) (Invitrogen, Catalog #26140-079), 0.1 mM non-essential amino acids (Invitrogen, Catalog #11140-050), 0.045 μg mL^−1^ of penicillin and 0.045 mg mL^−1^ of streptomycin antibiotics (penicillin–streptomycin liquid, Invitrogen, Catalog #15140-122) and sterilized using a filter (Corning, Catalog #431097). To pass the cells, the adherent culture was trypsinized with 0.25% Trypsin with EDTAX4Na (Invitrogen, Catalog #25200-114) and diluted in fresh medium upon reaching 50–90% confluence.

For transient transfections, ~150,000 cells in 1 mL of complete medium were plated into each well of 12-well culture treated plastic plates (Griener Bio-One, catalog #665180) and grown for 20–24 h. For JetPrime transfection, 500 ng of the plasmid and 250 ng of untranslatable “junk” DNA was added to 75 µL of JetPrime buffer and 1.75 µL JetPrime (Polyplus Transfection, catalog #114-15). Transfection solutions were mixed and incubated at room temperature for 10 min. The transfection mixture was then applied to the cells and mixed with the medium by gentle shaking. When applicable, doxycycline (Clontech, catalog #631311) was added immediately following transfection.

### Flow cytometry

Approximately 48 h post transfection cells from each well of the 12-well plates were trypsinized with 0.2 mL 0.25% Trypsin-EDTA at 37 °C for 3 min. Trypsin-EDTA was then neutralized by adding 0.7 mL of complete medium. The cell suspension was centrifuged at 4000 rpm for 1 min and after removal of supernatants, the cell pellets were resuspended in 0.5 mL PBS buffer (Dulbecco’s Phosphate Buffered Saline; Mediatech, catalog #21-030-CM). The cells were analyzed on a BD LSRFortessa flow analyzer. TagCFP was measured with a 445-nm laser and a 470/20 band-pass filter, mKate2 with a 561-nm laser, 600 emission filter and 610/20 band-pass filter, and YFP with a 488-nm laser, a 525 emission filter and 545/35 band-pass filter. For all experiments performed 100,000 events were collected. A FSC (forward scatter)/SSC (side scatter) gate was generated using a un-transfected negative sample and applied to all cell samples. We use a compensation matrix on our flow cytometry data to remove cross-talk observed between the three fluorescent proteins (Supp. Fig. 13). Data processing was performed in FlowJo 7.6.5. All experiments were performed in triplicates.

### Data availability

The data that supports the findings of this study are available from the corresponding author, L. B. (bleris@utdallas.edu), upon reasonable request.

## Electronic supplementary material

Supplementary Material

## Electronic supplementary material

**Supplementary information** accompanies the paper on the *npj Systems Biology and Applications* website (10.1038/s41540-017-0043-y).
