# Supplementary material for: Mapping the operational landscape of microRNAs in synthetic gene circuits
Source: NPJ Syst Biol Appl. 2018 Jan 11;4:6. doi: 10.1038/s41540-017-0043-y (PMC5765153; doi:10.1038/s41540-017-0043-y)
Supplement: Supplementary file 1 — Supplementary Material [file 41540_2017_43_MOESM1_ESM.docx]

**Supplementary Material**

**Mapping the operational landscape of microRNAs in synthetic gene circuits**

Tyler Quarton^1,2^, Kristina Ehrhardt^1,2^, James Lee^3^, Srijaa Kannan^4^, Yi Li^1,2^, Lan Ma^1^, Leonidas Bleris^1,2,3^

^1^ Bioengineering Department, University of Texas at Dallas, Richardson, Texas, USA

^2^ Center for Systems Biology, University of Texas at Dallas, Richardson, Texas, USA

^3^ Department of Biological Sciences, University of Texas at Dallas, Richardson, Texas, USA
^4^ School of Behavioral and Brain Sciences, University of Texas at Dallas, Richardson, Texas, USA

Correspondence should be addressed to L.B. ([bleris@utdallas.edu](mailto:bleris@utdallas.edu))


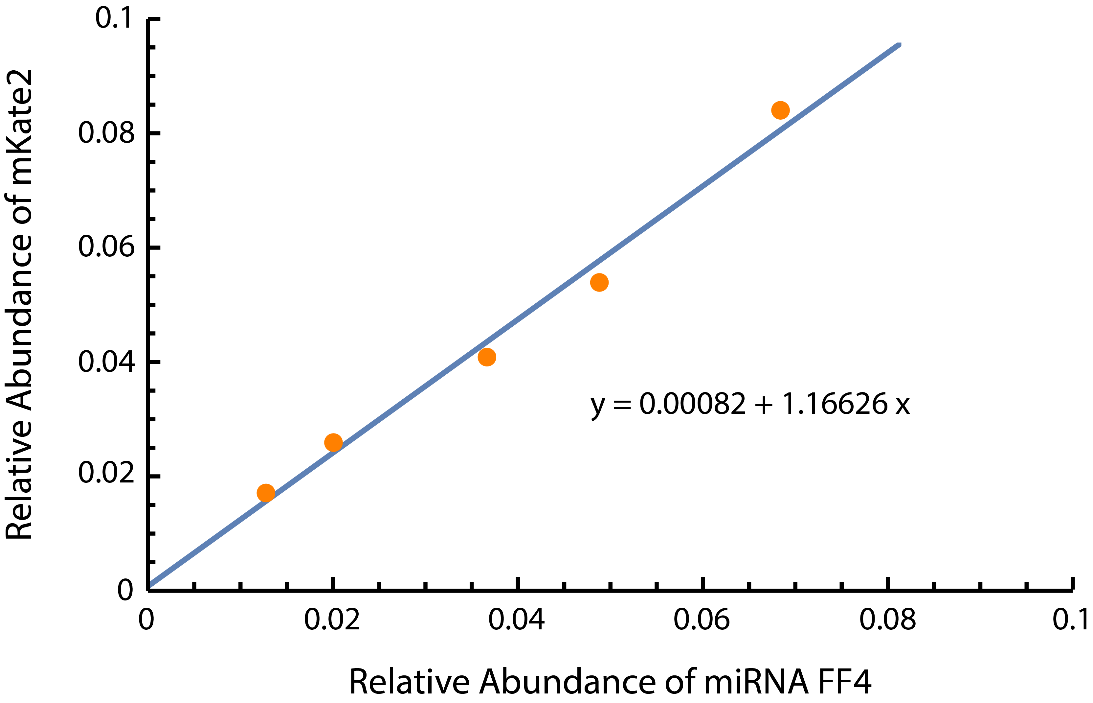


Supplementary Figure 1: Quantification of the relationship between mKate2 and miRNA-FF4. Quantitative results of qPCR measurements reveal a linear relationship between mKate2 and miRNA-FF4.

In order to establish the correlation between mKate2 and miRNA-FF4 we performed the following qPCR experiment: 31.25, 62.5, 125, 250 and 500 ng of the miRNA sensor plasmid was co-transfected with 100 ng of PCMV-RTTA into HEK293 cells. 1 μg/mL DOX was added. 48 hours later, the cells were harvested and the total RNA was harvested using the RNeasy Mini Kit (Qiagen, #74104). First strand synthesis was performed using the QuantiTect Reverse Transcription Kit (Qiagen, #205311). Quantitative PCR was performed using the KAPA SYBR FAST Universal qPCR Kit (KAPA Biosystems, #KK4601). GAPDH was used for normalization. The relative mRNA levels for mKate and miRNA were calculated using the 2^−ΔCt^ method.


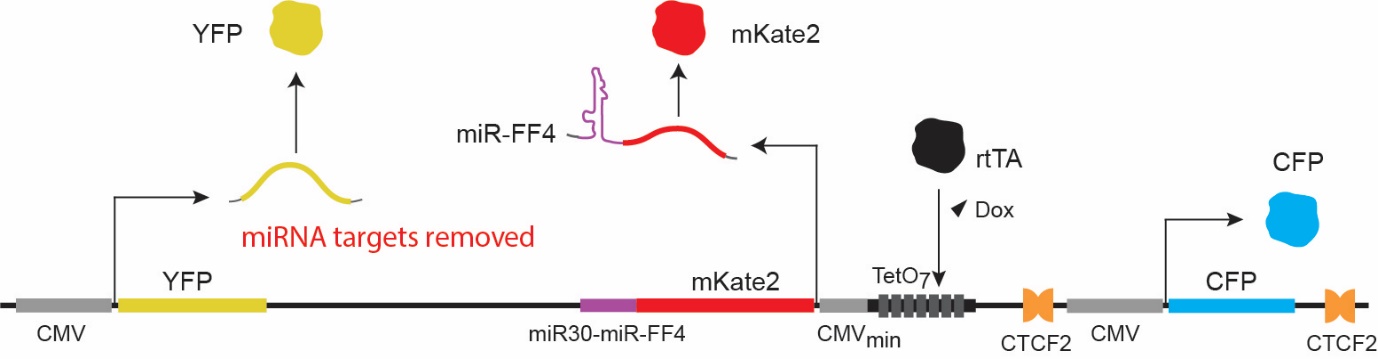


Supplementary Figure 2: Biological schematic of the control. The constitutively produced CFP fluorescent protein is used to quantify plasmid copy number. The synthetic miRNA-FF4 is produced in response to Dox and is quantified by mKate2 fluorescence. The FF4 targets found within the 3’UTR of the circuit’s output fluorescent protein YFP have been removed.


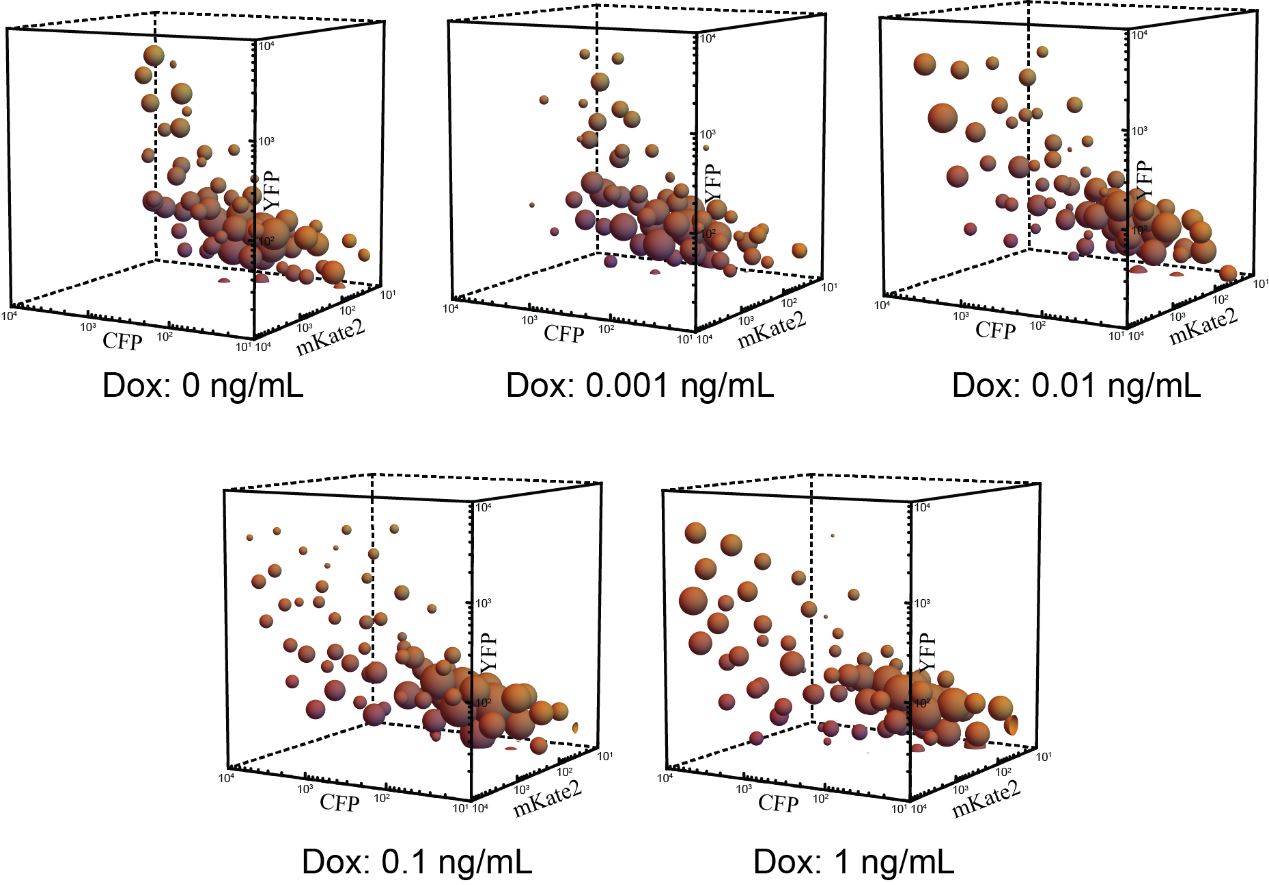


Supplementary Figure 3: Dox titration cluster plots of control. Cells containing the control were clustered using k-means clustering. The color of a cluster is a blend of yellow, red, and blue in proportion to the cluster’s mean values of YFP, mKate2, and CFP fluorescent arbitrary units, respectively. The diameter of a cluster is proportional to the number of cells it contains


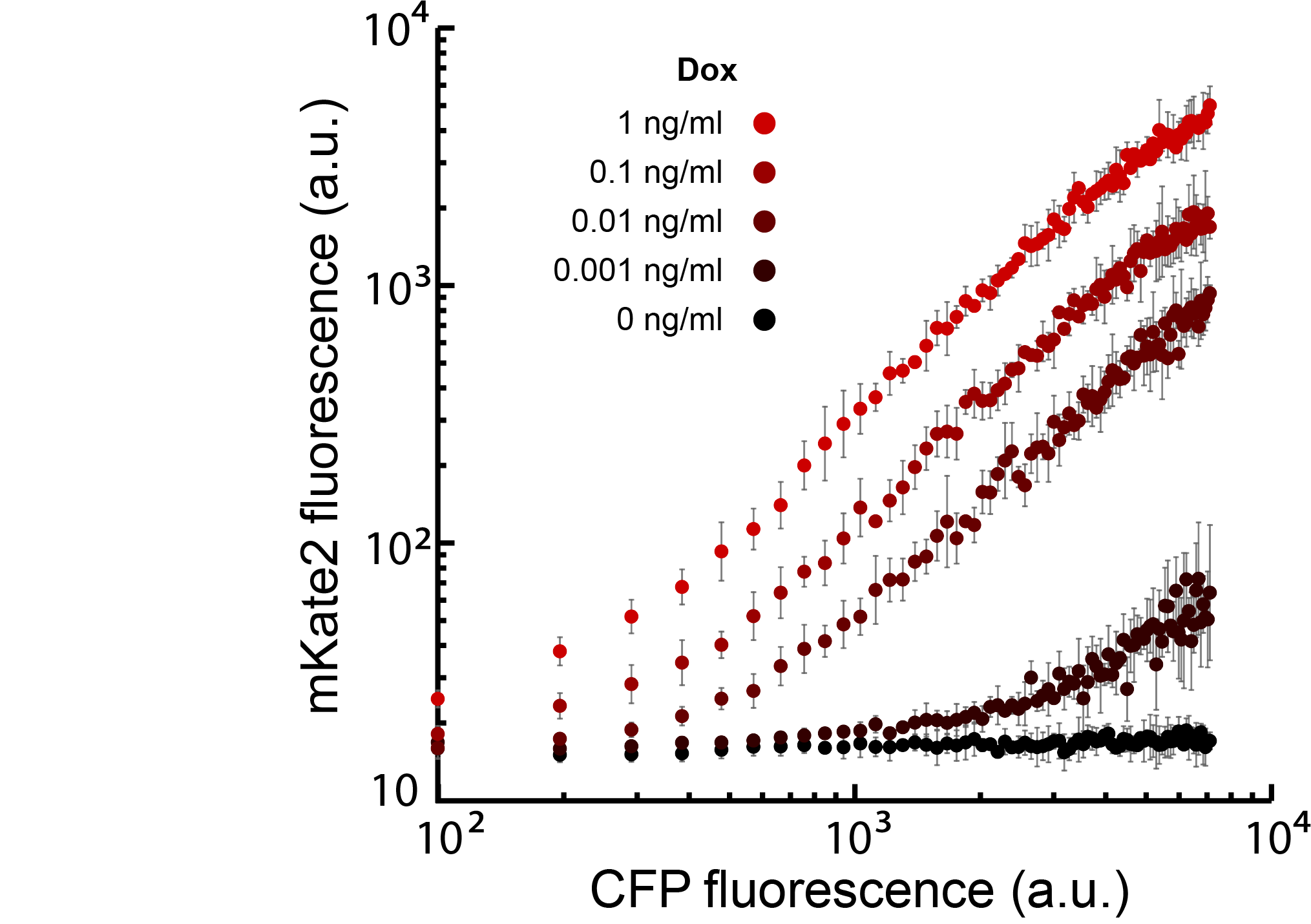


Supplementary Figure 4: mKate2 fluorescence of the control linearly increases as CFP increases in a Dox dependent manner.


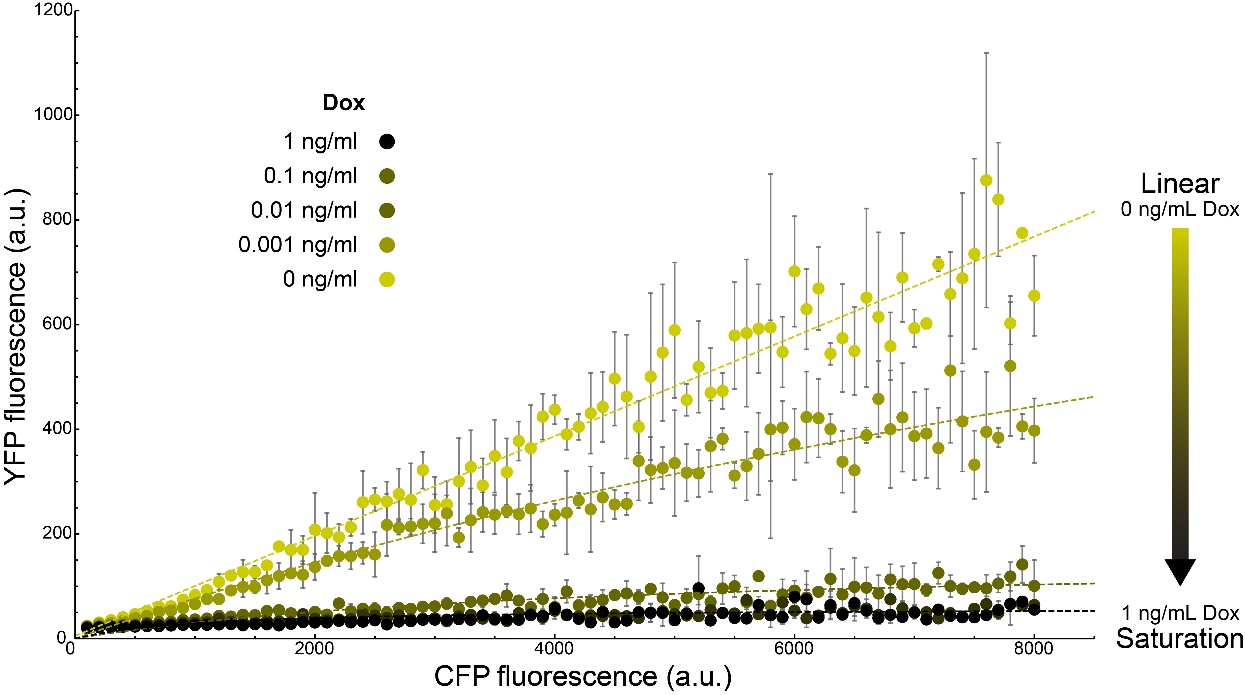


Supplementary Figure 5: Linear-linear plot perspective of YFP behavior as a function of CFP. YFP fluorescence transitions from a linear response to saturating response as a function of plasmid copy numbers at increasing Dox concentrations.


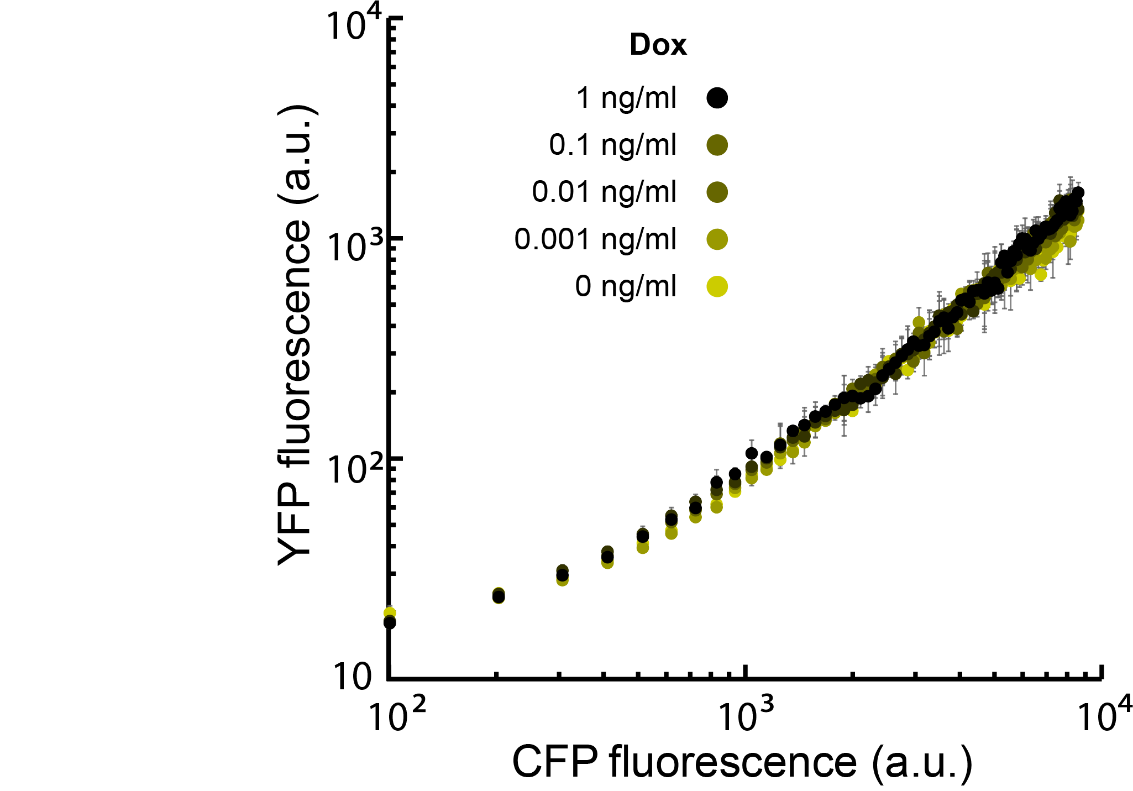


Supplementary Figure 6: Log-log plot perspective of control YFP fluorescence linearly responding to increasing CFP fluorescence independent of Dox.


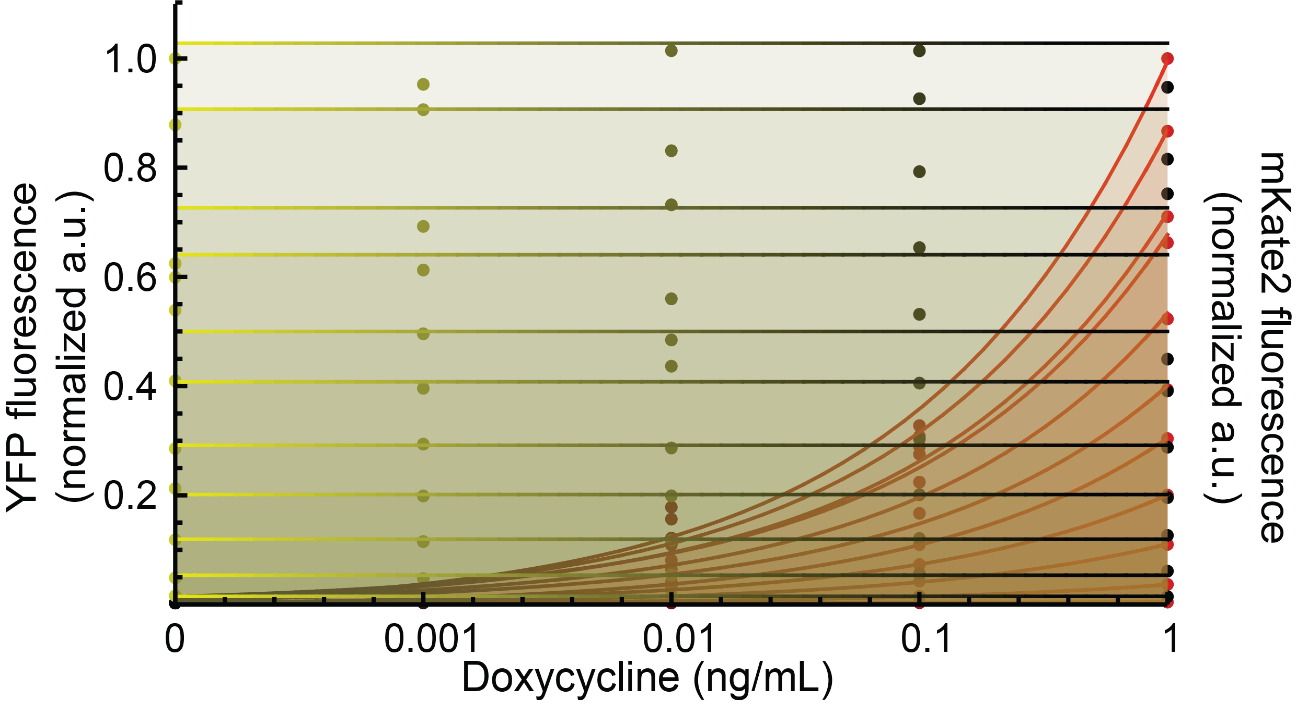


Supplementary Figure 7: Relative YFP expression of the control is unaffected by Dox whereas relative expression of mKate2 exponentially increases as a function of Dox.


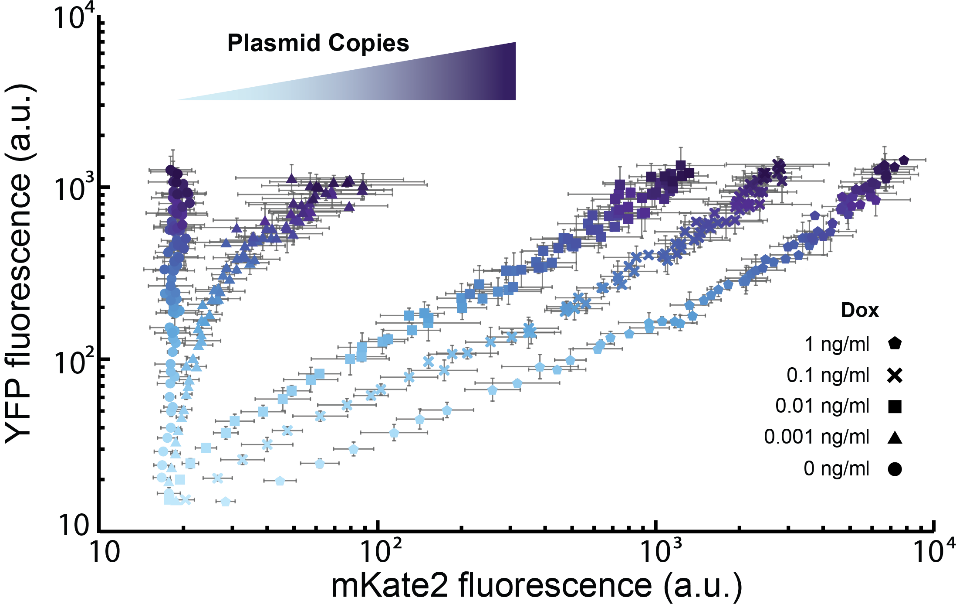


Supplementary Figure 8: The control’s YFP fluorescence is unaffected by increasing levels of Dox concentration at levels of CFP fluorescence whereas increasing Dox levels increases mKate2 fluorescence.


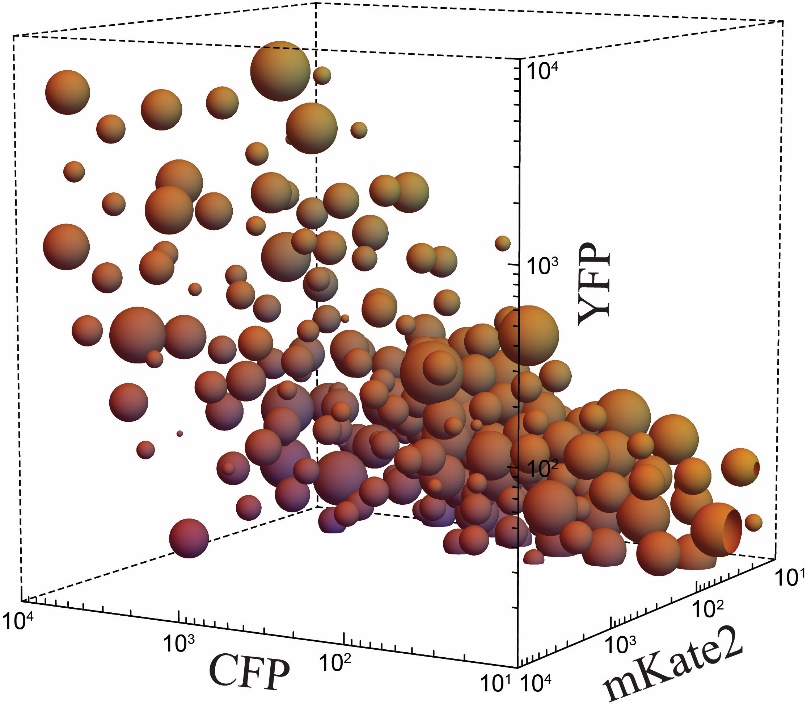


Supplementary Figure 9: Superposition of clustered data at each Dox concentration of cells containing the control circuit. The color of a cluster is a blend of yellow, red, and blue in proportion to the cluster’s mean values of YFP, mKate2, and CFP fluorescent arbitrary units, respectively. The diameter of a cluster is proportional to the number of cells it contains.


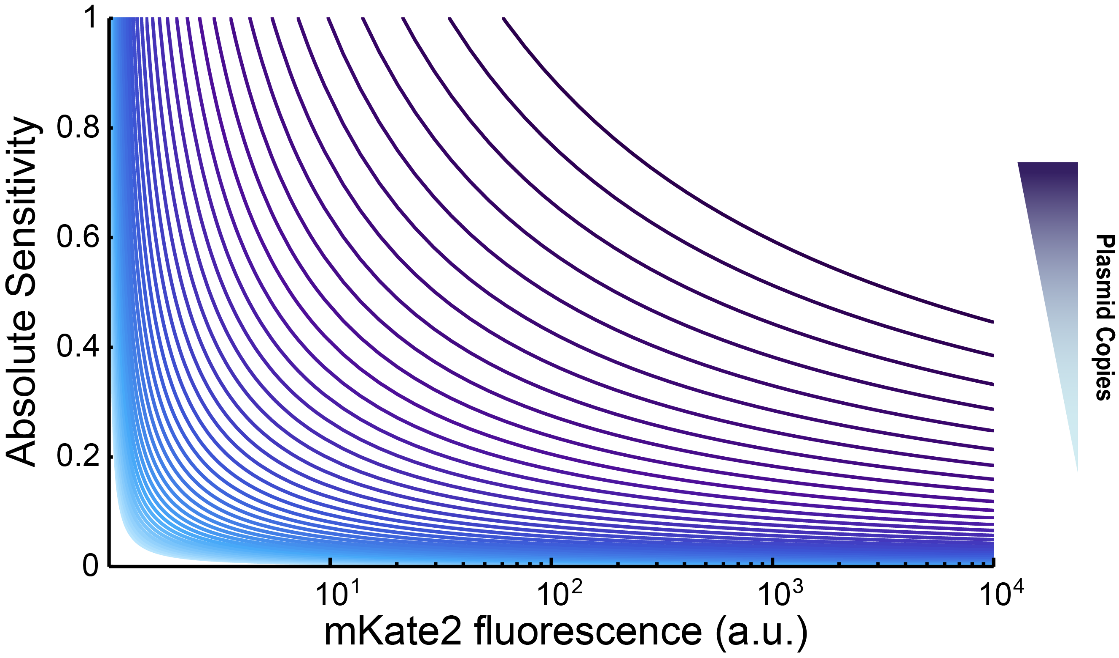


Supplementary Figure 10: Output sensitivity with respect to plasmid copies. As plasmid copies increases, the circuit output becomes increasingly sensitivity to mKate2 fluorescence.


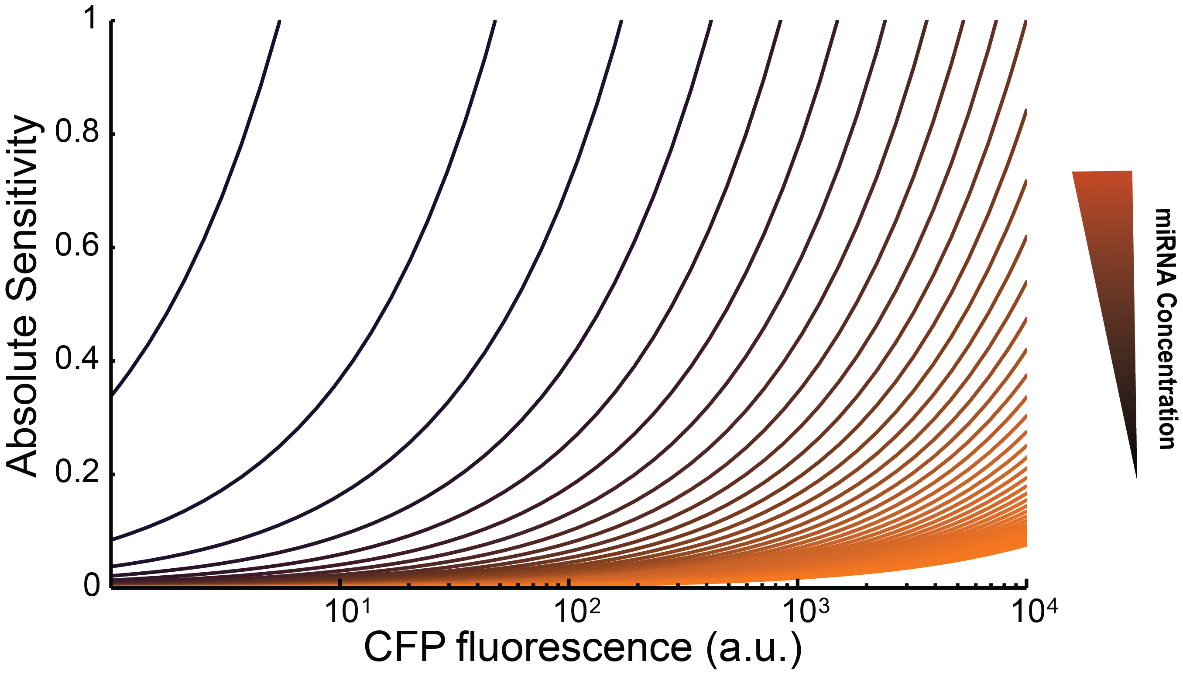


Supplementary Figure 11: Output sensitivity with respect to miRNA concentration. As miRNA concentration increases, the circuit output becomes increasingly robust to changes in CFP fluorescence.


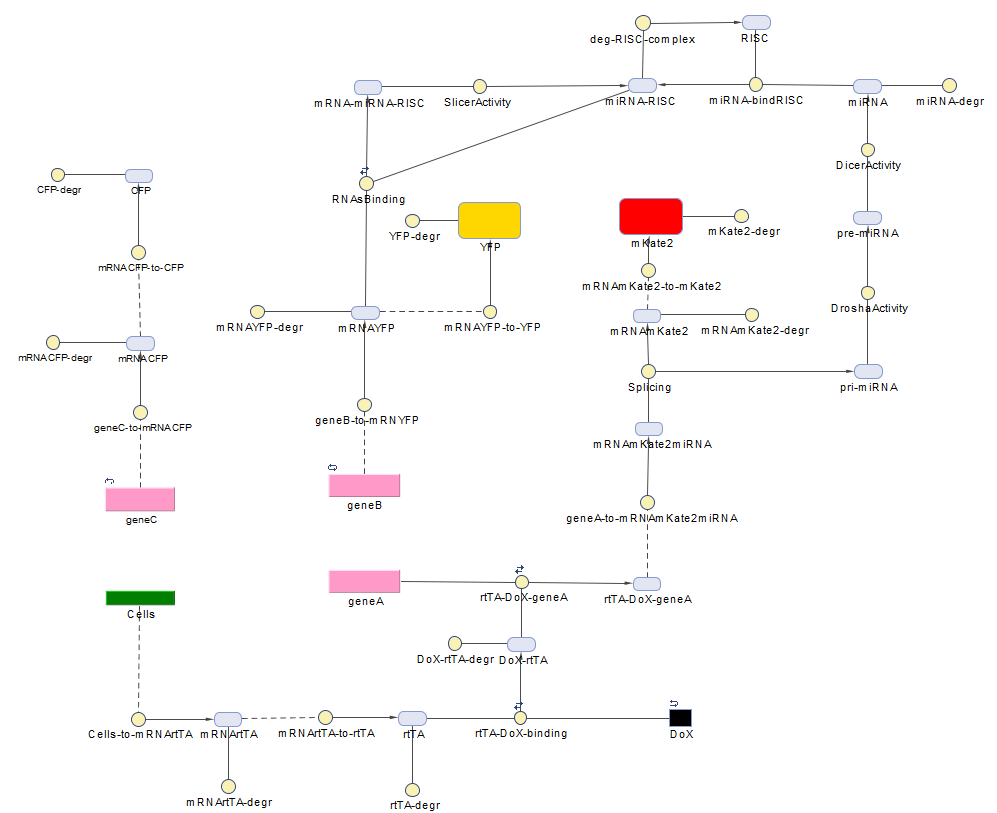


The above diagram corresponds to the following reaction array:

| YFP -> null |
| --- |
| mRNAYFP -> YFP + mRNAYFP |
| geneB -> mRNAYFP + geneB |
| [DoX-rtTA] -> null |
| rtTA -> null |
| DoX + rtTA <-> [DoX-rtTA] |
| [DoX-rtTA] + geneA <-> [rtTA-DoX-geneA] |
| mRNAYFP -> null |
| Cells -> Cells + mRNArtTA |
| mRNArtTA -> null |
| mRNArtTA -> mRNArtTA + rtTA |
| miRNA + RISC -> [miRNA-RISC] |
| [miRNA-RISC] -> RISC |
| miRNA -> null |
| mRNAmKate2 -> null |
| mKate2 -> null |
| [mRNA-miRNA-RISC] -> [miRNA-RISC] |
| [miRNA-RISC] + mRNAYFP <-> [mRNA-miRNA-RISC] |
| [pre-miRNA] -> miRNA |
| [pri-miRNA] -> [pre-miRNA] |
| mRNAmKate2miRNA -> [pri-miRNA] + mRNAmKate2 |
| mRNAmKate2 -> mRNAmKate2 + mKate2 |
| [rtTA-DoX-geneA] -> mRNAmKate2miRNA + [rtTA-DoX-geneA] |
| geneC -> mRNACFP + geneC |
| mRNACFP -> mRNACFP + CFP |
| CFP -> null |
| mRNACFP -> null |

The initial values of the species geneA, geneB, and geneC were scanned from values of 10 to 100 in steps of 1 simulating different plasmid copy numbers. A further scan of Dox was at each gene quantity was performed where the initial value of Dox was scanned from values of 0 to .9 in 10 steps of .1. Each simulation was run for $3\cdot{10}^{5}$ seconds upon which the proteins YFP, mKate2 and CFP reach steady-state. The steady-state values of each were then recorded and log transformed to correspond to our experimental data.


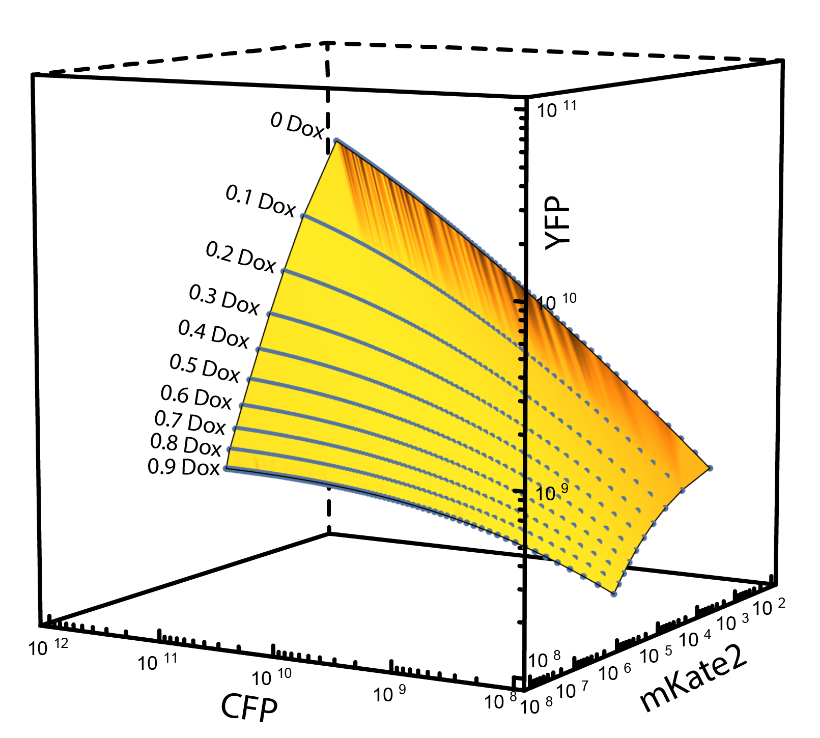


Supplementary Figure 12: Operational output space of circuit in silico. Simulated steady state values of each fluorescent protein were extracted from the above ordinary differential equation system at various Doxycycline concentrations.


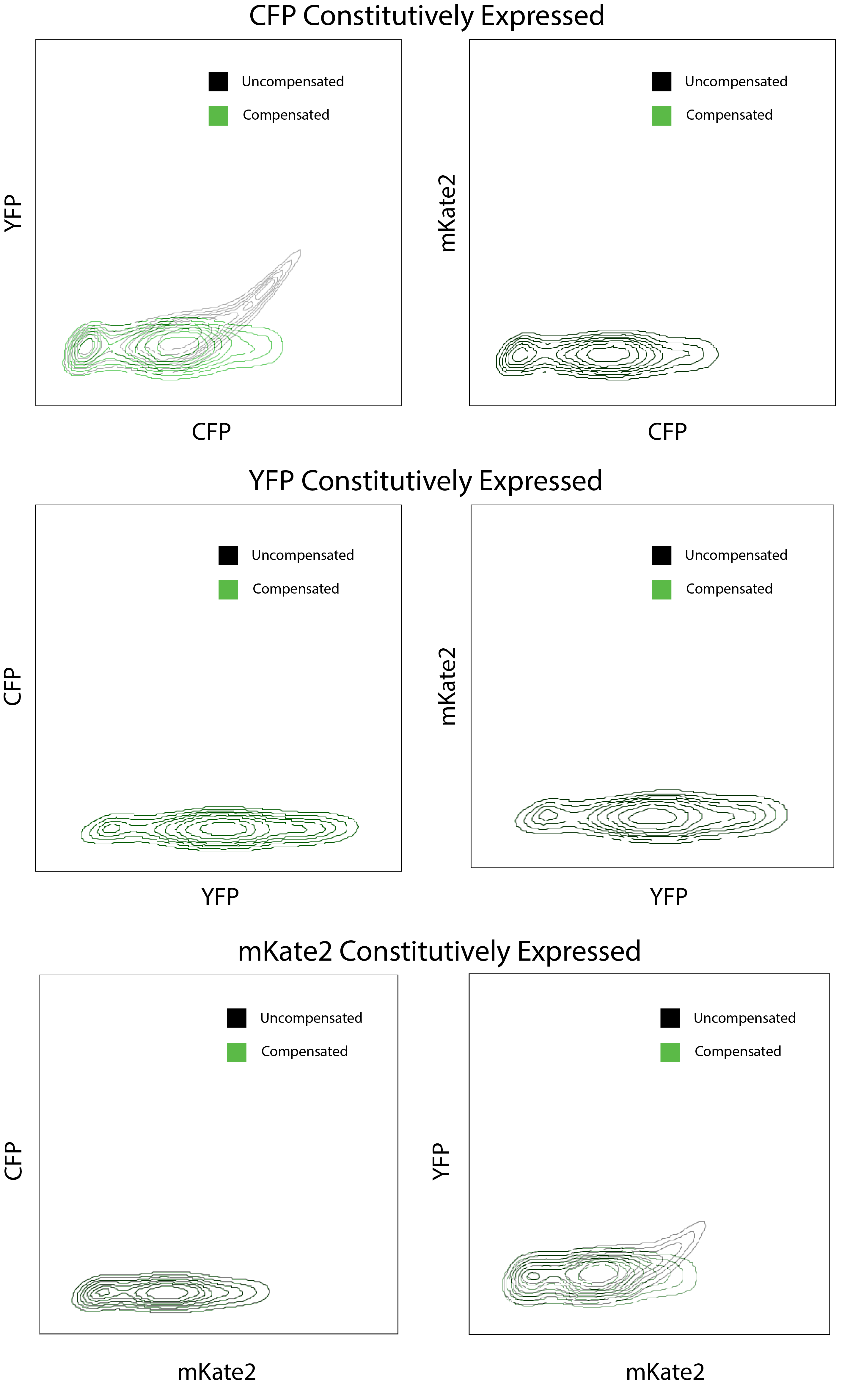


Supplementary Figure 13: Fluorescent emission cross-talk. Plasmids which express CFP, mKate2, and YFP were individually transfected in HEK-293 cells. The resulting data was used to calculate the compensation matrix applied to our data.

$$\begin{matrix} & \mathrm{YFP} & \mathrm{CFP} & mKate2 \\ \mathrm{YFP} & 100 & 0 & 0 \\ \mathrm{CFP} & 1.8 & 100 & 0 \\ mKate2 & 0.7 & 0 & 100 \end{matrix}$$
